# “Capacitive Sensor” to Measure Flow Electrification and Prevent Electrostatic Hazards

**DOI:** 10.3390/s121114315

**Published:** 2012-10-25

**Authors:** Thierry Paillat, Gerard Touchard, Yves Bertrand

**Affiliations:** 1 Electrofluidodynamic Group, P' Institute, CNRS, University of Poitiers, ENSMA, UPR 3346, 86962 Futuroscope Poitiers, France; E-Mail: gerard.touchard@univ-poitiers.fr; 2 Electricite de France (EDF), Site des Renardières LGEHT1, 77818 Morêt sur Loing, France; E-Mail: yves.bertrand@edf.fr

**Keywords:** flow electrification, electrostatic hazard, power transformer

## Abstract

At a solid/liquid interface, physico-chemical phenomena occur that lead to the separation of electrical charges, establishing a zone called electrical double layer. The convection of one part of these charges by the liquid flow is the cause of the flow electrification phenomenon which is suspected of being responsible of incidents in the industry. The P' Institute of Poitiers University and CNRS has developed an original sensor called “capacitive sensor” that allows the characterization of the mechanisms involved in the generation, accumulation and transfer of charges. As an example, this sensor included in the design of high power transformers, could easily show the evolution of electrostatic charge generation developed during the operating time of the transformer and, therefore, point out the operations leading to electrostatic hazards and, then, monitor the transformer to prevent such risks.

## 1. Introduction

When a liquid is in contact with a solid, a physical chemical phenomenon creates a charge separation area called the “electric double layer” which polarizes the solid/liquid interface. Electrically charged species of one sign appear on the solid surface while species of the opposite sign are distributed within the liquid. These areas are responsible for electrokinetic phenomena including the phenomenon of flow electrification. Often associated with a major risk in the industrial world this phenomenon still attracts a lot of attention. In fact, the flow electrification phenomenon could induce charge accumulation in a duct or in a tank, enough to produce catastrophic electrostatic discharges. For hydrocarbon liquids such as oils and gasoline, many accidents caused by this phenomenon have been identified [1–5].

For the last thirty years, flow electrification events have been suspected to be responsible for power transformer failures. Damage surveys revealed evidence of electrical discharges (electric “tree” paths, “worm holes”, presence of carbon …) on inner pressboards [6–10].

In transformers, the electrical current circulating in the windings generates heat. The dielectric liquid ensures the electrical insulation of coils and the cooling of the transformer through its forced circulation. The oil impregnated papers and pressboards are used to insulate and to support the copper windings. The oil flow at the pressboard surface leads, on the one hand, to a space charge in the oil which can relax in contact with grounded metallic walls, and, on the other hand, to a space charge in the pressboard which accumulates. In real transformers, the phenomenon is even more complex because the pressboard is porous and not homogenous but it is a combination of several components that may induce various physico-chemical reactions with the oil impurities. More, the aging of power transformer components (pressboard, oil, copper…), due to temperature and moisture, influences the flow electrification phenomena. Indeed, it seems that flow electrification might generate a surface charge, which would induce electrical discharges at the pressboard-oil interface enhancing the phenomenon. The results showed that the space charge density was multiplied by three or four when the pressboard had been degraded by electrical discharges. This conclusion was alarming because it seemed that a chain reaction might happen inside the power transformer, until its failure [11].

Nowadays, the Electrostatic Charging Tendency (ECT) measurement in the Westinghouse protocol [12–15] and, to a smaller extent, the continuous aging test for dielectric dissipation factor, or tanδ, are the most commonly applied measurements for operating transformer monitoring. Previous studies have shown that these two oil parameters are not really reliable with regard to the charging process [16]. When transformer oil seems doubtful given the results of both tests, a third diagnostic measurement is also recommended: the leakage current. It consists in measuring the current resulting from flow electrification, collected on the windings of the unloaded transformer. These standard measures provide a characterization of insulating material but do not allow evaluation of the electrostatic hazard.

An experimental sensor called the capacitive sensor has been developed for ten years in the P' Institute of Poitiers University (previously known as the LEA Laboratory) with the collaboration of EDF. This sensor is based on the accident analyses in high power transformers in which electric discharges were observed on pressboard surfaces in very well insulated parts of transformers.

## 2. Flow Electrification Phenomena

The origin of the charge separation at the solid/liquid interface is still not well understood. Specific adsorption, desorption, corrosion *etc.* are, in the current state of knowledge, hypothetical mechanisms that may explain the creation of charges. In hydrocarbon mixtures as insulating oils, chemical impurities (at a few ppm concentration) from the liquid would cause the phenomena. In any case, the physico-chemical reactions at the interface leads to a separation of electric charges, forming two oppositely charged areas: one in the solid and the other in the liquid, the electroneutrality of the solid/liquid couple is always preserved.

Different models can be used to describe the electrical double layer. By likening it to a charged capacitor, one plate being associated with the solid and the other plate in the liquid, Perrin and Helmholtz [17,18] proposed the first model. Although simplistic, it provides a fairly good picture of the electrical double layer, but does not explain the action of certain parameters such as impurity concentration on the electrification phenomenon. The most realistic model has been probably proposed by Stern [19], who introduced two sublayers into the liquid, the compact layer and the diffuse layer. More recently, different authors have completed the Stern model description with hydrated sublayer or overcharged boundary layer [20,21]. The compact layer extends from the interface over ten angstroms. The prevailing electrical forces maintain solidarity within the interface electric charges that form. Beyond the compact layer, the charges in the liquid form the diffuse layer. These charges are free to move under the effect of an external electric field or within a liquid flow. The carriage of the diffuse layer charges by a liquid flow leads to the flow electrification phenomenon. The charges of the diffuse layer are distributed in the volume of the liquid. Their concentration is maximum close to the interface and decreases exponentially into the volume of the liquid. The thickness of the diffused layer could be assimilated to the Debye length. Thus about 87% of the total charges in the liquid are contained in the diffuse layer [22]. If the solid is a dielectric or an insulated conductor, charges may accumulate.

In high power transformers, the forced oil flow leads to a charge accumulation on the pressboard surface that increases the local electrical potential. The capacitive sensor was developed for estimating the accumulated charges (directly correlated to the local potential) on a pressboard, or any other solid dielectric materials, for a given geometry and experimental protocol routinely applied. Moreover it allows one to analyze all the charge exchanges and movements, and the electric equilibrium.

## 3. Capacitive Sensor Description

The capacitive sensor has been tested in a flow loop facility developed in the laboratory [23].

### 3.1. The Loop

**Figure 1.**
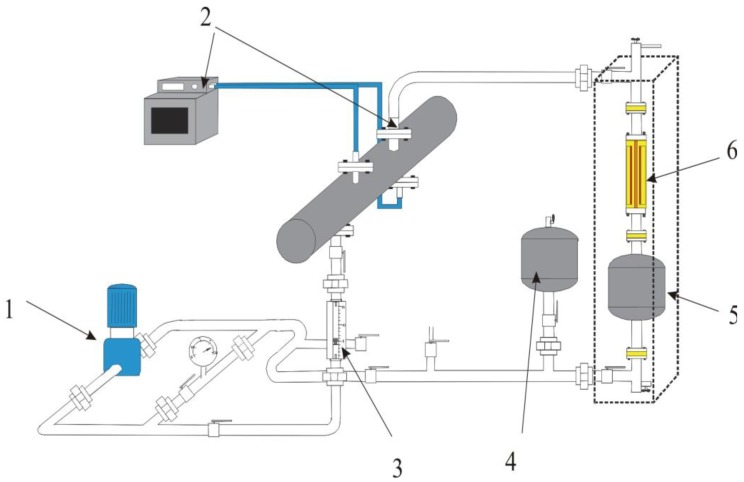
Test loop to simulate oil flow (1: pump, 2: heat regulation, 3: flow meter, 4: oil tank, 5: relaxation vessel, 6: capacitor sensor).

A stainless steel loop (Figure 1) was built to simulate the flow of oil along the pressboard used to support the copper windings of the transformer. The oil flow is induced by the pump (1) and controlled by the flow meter (2). It flows first in a heat exchanger (3) to acquire the desired temperature, then through the sensor (6), the electric relaxation vessel (5), and finally comes back to the pump. The reservoir (4) inserted into the flow loop allows the regulation of the static pressure of the liquid and also to fill the loop under vacuum. The humidity and the electrical conductivity of the oil are measured on line. 20 liters of oil are required to fill the setup. Because the measured currents are of the order of pico-amperes, several precautions were imposed on the loop to ensure the reproducibility of measurements:

- The loop is made exclusively of materials inert to mineral oils such as stainless steel, glass and viton^®^ in order to avoid any chemical reaction and oil pollution. Flow electrification phenomenon is quite sensitive to any pollution.
- The dimensioning of the pipes and the heat exchanger allows limiting the velocity of the oil. It is negligible compared to the oil flow velocity in the sensor. Thus, the oil entering the sensor can be considered completely electrically uncharged.
- The heat exchanger works thanks to a hot water circulation so without electrical perturbation (such as heat resistors) in the setup.

### 3.2. The Sensor

**Figure 2.**
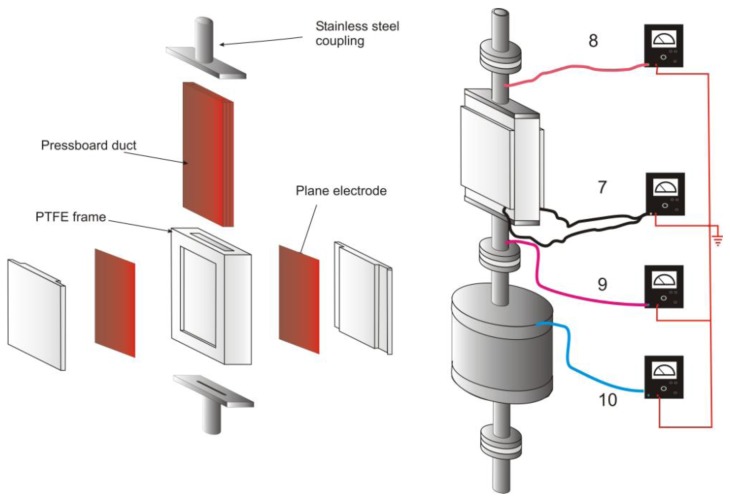
Capacitor sensor and current measurements: 7: accumulation current, 8: upstream leakage current, 9: downstream leakage current, 10: streaming current (generating current).

The sensor (Figure 2) consists of a rectangular pressboard channel (3 × 30 mm^2^ cross section, and 300 mm in length) made of 3 mm thickness pressboard sheets stuck together and inserted in a PTFE frame. Two stainless steel electrodes (240 × 50 mm) are placed facing the largest external surfaces of the pressboard duct embedded in the PTFE frame. 2 mm thickness of PTFE insulates the pressboard from the electrodes. Connected to a picometer (Keithley 610C), these electrodes allow measuring a capacitive current called the accumulation current (7) correlated to the charge trapped on the pressboard surface and to the local potential increase. This configuration intends to simulate the pressboard parts inside power transformers which have high leakage impedance having regard to the imposed potential (windings and tank). The increase of the electric potential favors the migration of trapped charges on the solid surface to the ground. The electrical leakages occur upstream and downstream of the PTFE frame towards the two stainless steel tubes. Insulated from the rest of the loop by PTFE flanges coupling, and connected to two ammeters, these tubes allow measuring two currents called respectively upstream and downstream leakage currents (8–9). Moreover, the liquid charges are carried by the flow into the relaxation vessel (Figure 1, number 5). According to the rates of oil flow and electrical properties of the oil, the reservoir volume of 10 L is sufficient to allow the complete relaxation of charges in the liquid. The resulting current, measured with a fourth pico-ammeters, is the streaming current also called the generating current (10). The dimensioning of the different parts of the loop is very sensitive and strict, especially the PTFE flanges. Thus, the thickness of the PTFE flanges must be sufficient to ensure the electrical insulation from the rest of the loop and not too thick to produce charges due to the oil flow on their surfaces. The geometry of the electrodes must allow distinction between the solid charges which leak at the solid/liquid interface to the ground direction, and the liquid charges carried by the flow. In the first version of the sensor only the accumulation current was measured. The interpretation of measurements gradually led to complete the loop with three additional current measurements.

### 3.3. Experimental Protocol

In order to allow a comparative analysis of all the oil/pressboard couples we studied with the sensor, the same experimental protocol is systematically applied. The loop and pressboard duct are dried by nitrogen gas flow before being submitted to vacuum (1.33 Pa) for 24 hours. The filling of the equipment is then done also under vacuum by direct transfer from commercial oil tanks. Finally oil flows in the loop for about 2 hours with a bulk pressure of 2,000 Pa to impregnate the pressboard duct. In addition, a 24 hours relaxation period is always applied before starting the experiment campaign.

The measurement sessions are planned on two or three consecutive days. They include an oil temperature cycle (20–10–20–40–60–80–20 °C) representative of different operating conditions of the transformer: start-up, operation, over-heating. More, this temperature cycle allows observing evolution of parameters after heating and cooling. For each temperature, three laminar oil flow rates (132, 220, and 308 L/h), leading respectively to mean velocities of 40, 68 and 95 cm/s in the pressboard duct are tested.

### 3.4. Oil/Pressboard Couples

Over 100 oil/pressboard pairs have been assessed on the sensor with different objectives:

- Qualification of new products (“standard pair”): oils (or, to a lesser extent, pressboards) proposed by the oil industries are constantly changing their chemical composition for economic and technical reasons. Some oils are disappearing as new products are marketed. All these developments require determining the physical properties of these oils, such as electrostatic behavior, before use in power transformers.
- Monitoring the aging and maintenance of operating transformers (“standard pair”): the evolution over time of dielectric materials may require technical operations such as oil reconditioning or regeneration, topping up the oil level in the transformer or more simply to change the oil. The oil testing into the loop can then assess the impact of these operations on its electrostatic behavior.
- Screening of transformers under suspicion (“suspect pair”): specific patterns of dissolved gases in oil, electric discharge activities (detected by acoustic sensors) are all factors suggesting an electrostatic hazard in a transformer. Some pressboards and oils coming from these transformers have been studied for contribution to their supervision. In what follows, an oil/pressboard couple will be qualified as “suspect pair” if at least one of the two dielectrics is coming from a suspect transformer.
- Tools for studies of electrostatic behavior (“research pair”): the chemistry of pressboards and oils plays an important role in the electrostatic risk. In order to study it, pressboards whose chemistry is perfectly controlled and specially designed, and oils containing additives such as inhibitors and BTA, have been studied in the loop for the understanding of electrostatic mechanisms.

## 4. Experimental Measurement

### 4.1. Current Measurements

**Figure 3.**
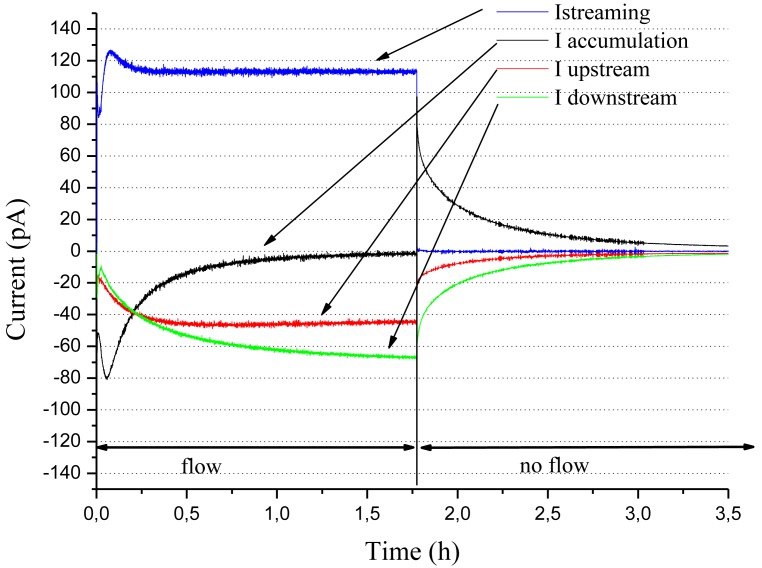
Typical currents measured on the sensor for “low leakage impedance” configuration (flow rate 308 L/h, temperature 40 °C).

Figure 3 shows a typical evolution of current measurements *versus* time. The sign of the currents is representative of the majority of the different mineral oil/pressboard pairs studied. With the exception of a few pairs, including silicone oils for example, currents related to pressboard charges (accumulation current, upstream and downstream leakage currents) are negative, the current associated with the oil (streaming current) is positive.

As soon as the oil starts to flow, a charge is generated at the interface. Positive charges in the oil are carried away by the flow and induce the streaming current which passes by a maximum value before reaching a steady state. In the same time, opposite charges are trapped inside the solid pressboard. The magnitude of the accumulation current increases and comes back more or less rapidly to zero. The surface potential increases, until reaching a steady state leakage current toward the grounded outlet and inlet ducts through paths along the interface. The dynamic of the transient state, the magnitude of the streaming current and the maximum value of the accumulation current depend on the oil/pressboard pair. When the oil flow is stopped, the continuous charge separation process stops at the interface and all solid charges leak to the ground. Accumulation current is then positive with negative leakage currents. It can be checked that the sum of the leakage (inlet and outlet), accumulation and streaming currents is equal to zero at all time.

Characteristic parameters considered as relevant with regard to flow electrification have been measured for several combinations of (new and used) oil/pressboard pairs. They depend on the studied pair, oil temperature and flow velocity. The principal studied parameters are:

- Charge accumulation “Q”, obtained from integration *versus* infinite time of the accumulation current with oil flow.
- Steady state streaming current.

### 4.2. Electrical Analogy

**Figure 4.**
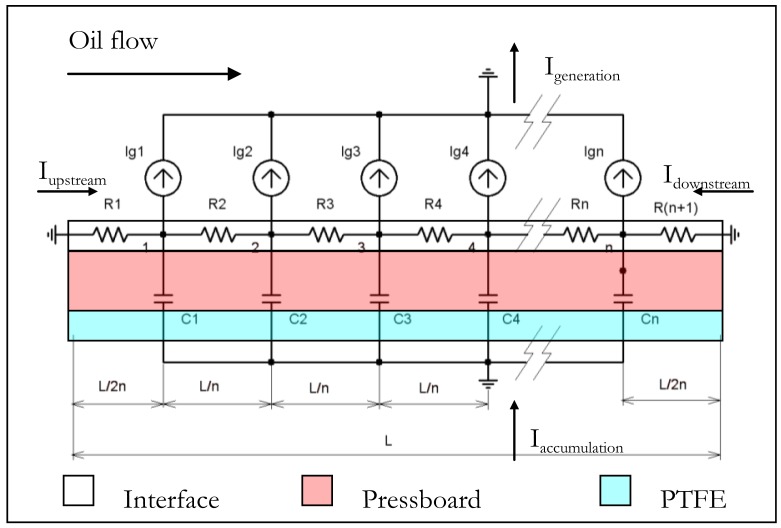
Electric equivalent circuit.

The electrical equivalent circuit allows representing this measurement system (Figure 4) [24,25]. The physicochemical phenomena at the interface which create the charges on the solid act as current generators distributed along the solid/liquid interface. The streaming current due to the convection of the liquid charges is equal but opposite of the current generation. The magnitude of these generating currents is decreasing from the duct entry due to the electrical double layer development along the interface. Resistor components are correlated to charge leakage in inlet and outlet pressboard duct. The resistor interface is linked to the electrical properties of the solid/liquid interface modified by electrical double layer charges. Finally, capacitors are associated to the electrical permittivity of both the solid (pressboard, PTFE, electrical cable) and the liquid. They are considered as constant along the duct.

Considering this circuit, the solid potential directly proportional to the solid accumulation charges depends on the generating current magnitude and the electrical properties of the solid/liquid couple.

## 5. Results and Discussions

After 10 years of tests and over hundred pairs of oil/pressboard expertise, the sensor has never allowed the production of electrostatic discharges and to reproduce the electrostatic hazards. The dimension of the sensor relative to power transformers is probably at the origin.

**Figure 5.**
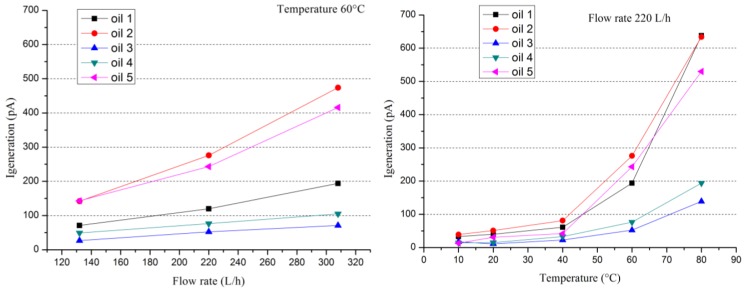
Current generator behaviors *versus* flow rate and temperature for different new oils flowing on the same pressboard.

**Figure 6.**
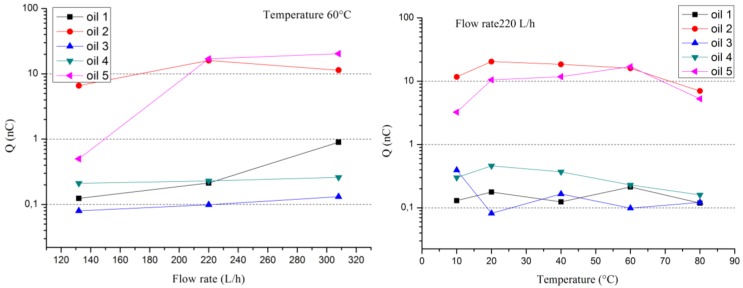
Charge accumulation behaviors *versus* flow rate and temperature for different new oils flowing on the same pressboard.

The potential on the surface pressboard (charge accumulation) is probably limited by the electrical insulation of the sensor, which is much lower than that encountered in practice. Anyway, the device allows comparing different oil/pressboard couples (Figures 5 and 6) in term of the phenomenon of flow electrification. These measurements could be analyzed according oil properties presented in Table 1.

Figures 5 and 6 present examples of the evolution of the current generation (or streaming current) and the charge accumulation *versus* flow rate, as well as temperature for different new oils and the same pressboard, respectively. Current generation behaviors are quite classical. They increase with flow rate and temperature. The current varies globally between 10 and 1,000 pA with some exceptional lower or higher values. For charge accumulation, the behavior is more complex. In fact, if the charge accumulation increases with the flow rate for all the different oil/pressboard studied *versus* temperature it is not foreseeable. It could increase, decrease or pass by a maximum or minimum. In fact, the charge accumulation is link to antagonist effects. The temperature raising favored the charge generation process at the interface but, at the same time, it increases the oil electrical conductivity and the charge leakage. Thus, the magnitude of the charge accumulation fluctuates over three decades of 0.1 nC to 100 nC.

In the past reducing the oil flow rate has been one answer to the electrostatic hazard in high power transformers. However, its efficiency is not so evident considering the behavior of charge accumulation due to the increasing of oil temperature which inevitably occurs with flow rate reduction. And moreover, it does not also consider the premature aging of the cellulose and other materials due to higher temperature resulting of lower cooling.

**Figure 7.**
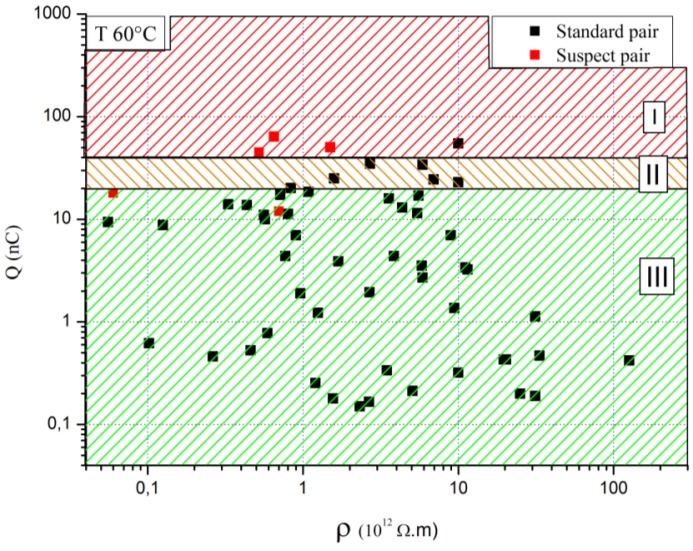
Charge accumulation on the pressboard surface *versus* oils resistivity (temperature 60 °C, flow rate 220 L/h). I: Suspicious Area; II: Intermediate Area; III: Safe Operation Area.

Charge accumulation measurements for standard pairs and suspect pairs *versus* oil resistivity for one flow rate and one temperature are presented in Figure 7[26]. The most interesting thing in this figure is the charge accumulation values for suspect pairs which are among the highest. The criterion of the accumulated charge seems to be satisfactory to identify a dangerous situation in a power transformer. However a suspect pair may lead to a high value of accumulated charge but without reaching a noteworthy one. Such a pair consists of oil from a transformer where an abnormal hydrogen content was observed. It remained in operation for several years until its withdrawal from the electricity network. On this basis, a graph of hazard assessment (Figure 7) was developed from the values of accumulated charge and oil resistivity. It considers three areas regardless of oil resistivity. An area described as suspicious (I), since in this area the transformer has a risk of developing an electrostatic activity. An area where the operation (III) of the transformer is quite safe with respect to electrostatic hazards. And finally between the two, an intermediate area (II) where measurements may differ whether the oil/pressboard pair is new or used. Statistically, we have shown that oils along time generally undergo an increase of the accumulated charge. Thus, the accumulated charge of a new oil, which had an initial value included in Zone II, may become classified risky with aging, while for a used oil, the value is acceptable. To build this graph, two choices were made and supported, on the one hand the shape of the separation areas, on the other hand the threshold values of these areas. They are justified by statistical analyses and the literature [27,28].

## 6. Conclusions

Thanks to this capacitive sensor, the measurement of the accumulated charge allows assessment of power transformer behaviour towards flow electrification and electrostatic hazard. Such a sensor could be easily re-designed to be implemented on the oil circuit of a real high power transformer in order to monitor online the flow electrification hazards in the transformer.

## Figures and Tables

**Table 1.** Oil properties associated to Figures 5 and 6 at 20 °C.

	**Oil Conductivity (pS/m)**	**Oil Moisture (ppm)**
Naphthenic Oil 1	0.11	3.9
Naphthenic Oil 2	0.01	2.4
Naphthenic Oil 3	0.01	5.5
Paraffinic Oil 4	0.50	3.0
Paraffinic Oil 5	0.23	4.1

## References

[1] Klinkerberg A., van der Minne J.L. (1958). Electrostatics in Petroleum Industry: The Prevention of Explosion Hazards.

[2] Luttens G., Wilson N. (1997). Electrostatic Hazards.

[3] Gavis J. (1968). Electric Charge Generation during Flow of Hydrocarbons through Microporous Media. Chem. Eng. Sci..

[4] Gibbings J. (1970). Electrostatic Charging in the Laminar Flow in Pipes of Varying Lengths. J. Electroanal. Chem..

[5] Walmsley H., Woodford G. (1981). The Generation of Electric Currents by the Laminar Flow of Dielectric Liquids. J. Phys. D Appl. Phys..

[6] Ieda M., Goto K., Okubo H., Miyamoto T., Tskioka H., Kohno Y. (1988). Suppression of Static Electrification of Insulated Oil for Large Power Transformers. IEEE Trans. Electr. Insul..

[7] Oommen T.V. (1988). Static Electrification Properties of Transformer Oil. IEEE Trans. Electr. Insul..

[8] Ieda M., Yanari T., Miyamoto T., Higaki H. Investigation of Static Electrification in Large Power Transformers in Japan.

[9] Lee M.J., Nelson J.K., Franchek M.A. Electrification in Transformer Model Structures.

[10] Lindgren S.R., Wahabaugh A.P., von Guggenberg P.A., Zahn M., Brubaker M., Nelson J.K. Temperature and Moisture Transient Effects on Flow Electrification in Power Transformer.

[11] Moreau E., Paillat T., Touchard G. Evolution of the Streaming Current Generated by an Oil Flow through a Pressboard—Application to Flow Electrification in Power Transformers.

[12] Oommen T.V., Petrie E.M. (1984). Electrostatic Charging Tendency of Transformer Oils. IEEE Trans. Power Appar. Syst..

[13] Perrier C., Beroual A. (2009). Experimental Investigations on Insulating Liquids for Power Transformers: Mineral, Ester and Silicone Oils. IEEE Electr. Insul. Mag..

[14] Mas P., Paillat T., Touchard G., Moreau O. (2000). Investigation in Order to Improve the E.C.T. Measurement Protocol of Large Power Transformer Oil. CEIDP.

[15] Palmer J.A., Nelson J.K. (1996). Method for Repetitive Measurement of the Electrostatic Charging Tendency of Liquid Dielectrics. CEIDP.

[16] Paillat T., Touchard G., Moreau O. Flow Electrification in Transformers: Correlation between Winding Leakage Current and Pressboard Charge Accumulation.

[17] Perrin J. (1904). Mécanisme de l'électrisation de contact et solution colloïdale. J. Chim. Phys..

[18] Helmholtz H. (1879). Studien über electrische Grenzschichten. Ann. Phys. Chem..

[19] Stern O. (1924). Zur Theorie der Electrolytischen. Z. Elektrochem..

[20] Grahame D.C. (1947). The Electrical Double Layer and the Theory of Electrocapilarity. Chem. Rev..

[21] Zmarzly D. (2009). Streaming Electrification Current Density Distribution inside Pipes Assuming Overcharged Boundary Layer. IEEE Trans. Dielectr. Electr. Insul..

[22] Vazquez-Garcia J., Rivenc J., Agneray A., Paillat T., Touchard G. (2005). A Critical Approach to Measure Streaming Current: Case of Fuel Flowing through Conductive and Insulting Polymers Pipes. IEEE Trans. Ind. Appl..

[23] Moreau O., Paillat T., Touchard G. (2004). Flow Electrification in Transformers: Sensor Prototype for Electrostatic Hazard. Electrostatics 2003.

[24] Cabaleiro J.M., Paillat T., Moreau O., Touchard G. (2009). Electrical Double Layer's Development Analysis: Application to Flow Electrification in Power Transformers. IEEE Trans. Ind. Appl..

[25] Paillat T., Moreau O., Cabaleiro J.M., Perisse F., Touchard G. (2006). Electrisation par écoulement modélisation électrique. J. Electrost..

[26] Paillat T., Morin G., Touchard G., Bertrand Y., Moreau O., Tanguy A Electrostatic Hazard in High Power Transformers: Analyze of Ten Years of the Capacitive Sensor.

[27] Masanori K., Shuhei K., Shigemistu O., Tsuyoshi A. (2009). Aging Effect on Electrical Characteristics of Insulating oil in Field Transformers. IEEE Trans. Dielectr. Electr. Insul..

[28] Hebner R.E., Kelley E.F., Forster E.O., Fitzpatrick G.J. (1985). Observation of Prebreakdown and Breakdown in Liquid Hydrocarbons. IEEE Trans. Electr. Insul..

